# Behcet's disease presenting with primary hypothyroidism, adrenal insufficiency, and celiac disease: A case report

**DOI:** 10.1002/ccr3.3097

**Published:** 2020-07-14

**Authors:** Ameer Kakaje, Rama Awad

**Affiliations:** ^1^ Faculty of medicine Damascus University Damascus Syria; ^2^ Department of endocrinology Al Mouwasat University Hospital Damascus University Damascus Syria

**Keywords:** Addison's disease, Behcet's disease, celiac disease, Crohn's disease, hypothyroidism, polyglandular autoimmune syndrome, immune‐modulating drugs

## Abstract

We are reporting a case report of Behcet's disease with autoimmune dysfunction that affected the thyroid and adrenal glands and the patient also had sub‐clinical celiac disease. However, the correlation between Bechet's disease and autoimmune disease is still controversial.

## Graphical abstract

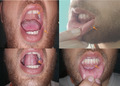

## 1 INTRODUCTION

Behcet's disease (BD) is a type of vasculitis that is characterized by periods of aggravations and improvements. Behcet's disease presents is a chronic inflammation that affects multiple systems and vessels in a young patient.^1^ Behcet's disease does not have a clear definition as the etiology is unknown, and it influences multiple organs.^2^ This study presents patient with BD and has multiple autoimmune diseases (AIDs) which may suggest a common etiology.

## 2 CASE REPORT

A 21‐year‐old man came to the emergency department (ED) complaining of nausea and recurrent vomiting for the past 2 days. He also suffered for a month from malaise and dizziness when standing up which improved when lying down. No loss of consciousness, diarrhea, or haematemesis was reported. He had a history of BD which was diagnosed 4 years ago. At that time, he had arthralgia and presented with the symptoms of BD triad, which included recurrent oral aphthous and genital ulcers, and iritis. Subsequently, the patient was put on cyclosporine and naproxen, but decided to stop the treatment when symptoms improved which was 18 months before presenting again at the ED. He denied taking cortisol and had no significant surgical history. Family history revealed that the brother had Crohn's disease and the sister had rheumatoid arthritis. The patient's parents did not have consanguineous marriage.

**Figure 1:**
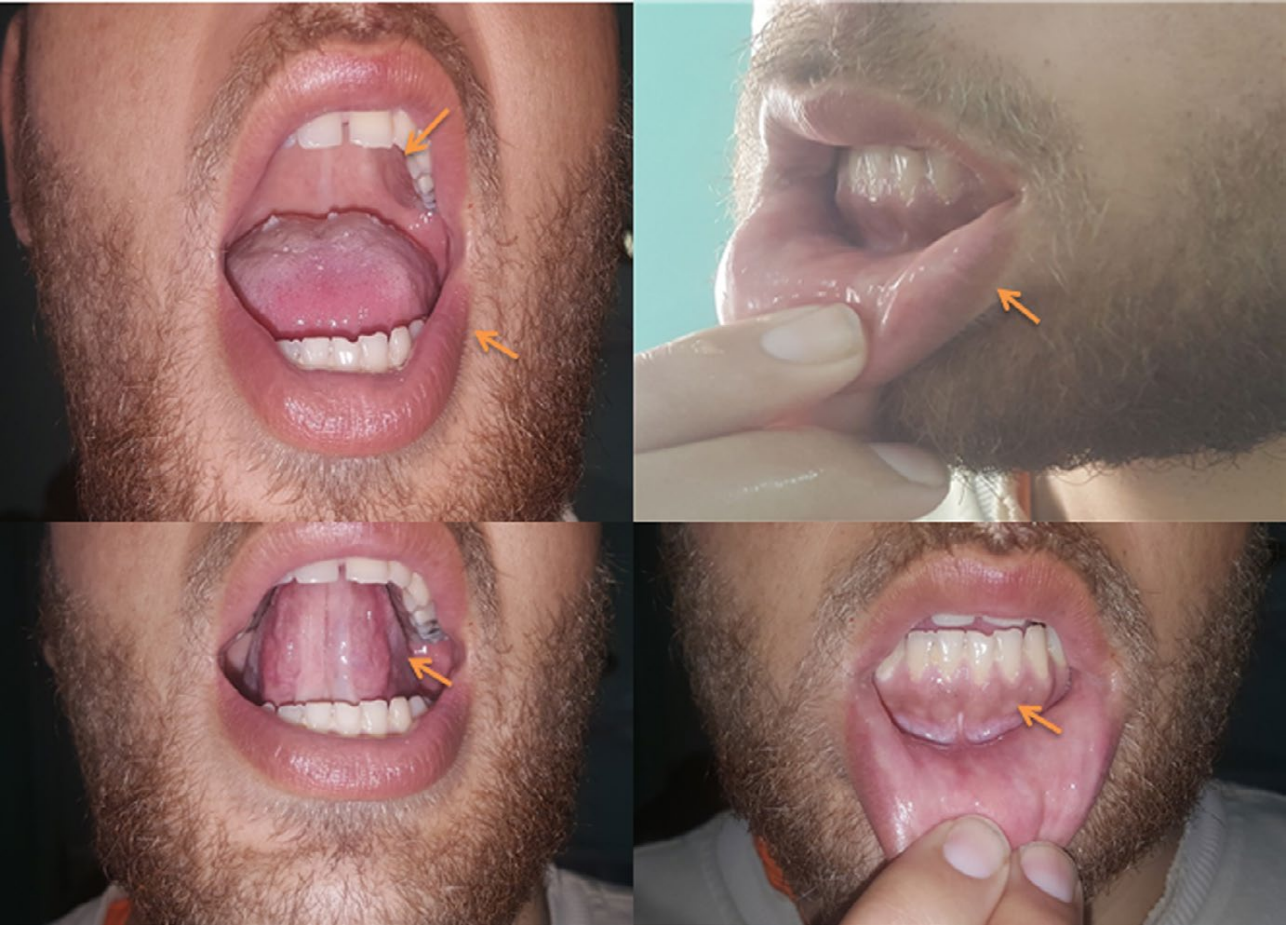
Shows oral hyperpigmentation (orange arrows)

On examination, the patient was afebrile. Blood pressure (BP) was low (60\40 mm Hg), and the patient had tachycardia (117 beats/min). The patient reported normal appetite and denied weight loss. Hyperpigmentation was observed in the internal mucosa of the lips and gingiva (Figure 1). Normal saline was infused and anti‐emetics were given intramuscularly. Afterward, BP improved (100\70 mm Hg) and heart rate went back to normal range. His laboratories showed hyponatremia, mild hyperkalemia, and normal glucose levels. ECG and chest x‐ray were also normal. Morning 8 am cortisol was low with a high ACTH level which indicated adrenal insufficiency of Addison's disease (AD). Furthermore, TSH level was elevated, and TPO antibodies were positive. Thyroid ultrasonography showed a diffuse enlarged thyroid gland. Nuclear imaging showed low iodine uptake in the thyroid. This resulted in the diagnosis of Hashimoto's disease (HD). Anti TTG IgA level was high and duodenum biopsy by endoscopy showed stage 3a Marsh score which indicated an underlying celiac disease (CD).

## 3 DISCUSSION

The patient in this study presented with BD triad leading to the diagnosis of BD in accordance with ICBD 2014 criteria.^3^ Behcet's disease lacks specific histological and laboratory findings,^2^ has polymorphous features, and can be fatal if left untreated. Behcet's disease etiology and pathophysiology are controversial with conflicting data; it is believed that BD disturbs the regulation of immune system, which increases the tendency of acquiring another AID.^4^ Behcet's disease might also resemble Crohn's disease as they both have polygenic auto‐inflammatory factors where IL‐1β increases.^5^, ^6^ Therefore, having a sibling with Crohn's disease might be a risk factor.

By contrast, BD typically causes hyper‐ vascularization in the majority of endocrine glands and can trigger vasculitis. Therefore, autoimmune etiology is speculated to be the main underlying cause for BD.^7^ Moreover, auto‐reactive T cells are suggested to cause the immunological disturbances of BD, resembling thyroid autoimmune dysfunction ^8^ which could explain the underlying correlation between HD and BD in our case. However, the exact effect that BD has on thyroid functions (TSH, fT4, and T3) is still unclear in the literature.

Furthermore, few studies correlate BD with AD. One study found partially dysfunctional adrenal glands in BD patients.^9^ Marsh score is a pathological assessment of the intestinal wall and is one of the criteria to diagnose CD along with positive serology. 3a Marsh score is characterized by crypt hyperplasia and mild atrophy of the villi and therefore our patient tested positive for CD despite having no symptoms. In this study, serology was positive and the biopsy of the duodenum showed atrophied intestinal villi with a 3a Marsh score which were suggestive of CD.

Polyglandular autoimmune syndrome (PGA) is hereditary immune dysfunction which affects multiple endocrine glands. PGA‐ll is the most common type and it has AD and autoimmune thyroid disease, diabetes mellitus type 1, or both and might have other AIDs like CD. This combination is also called “Schmidt syndrome”.^10^ In this case, the patient did not undertake genetic testing due to financial difficulties, but the family history was negative for HD, AD, type 1 diabetes, BD, and CD despite having 4 siblings. This suggests that hereditary PGA‐ll was highly unlikely. In this study, our patient suffered from multiple auto immune diseases including HD, AD, and CD along with BD. Moreover, it was not evident whether ceasing immune‐modulating drugs predisposed the patient to AIDs or caused PGA‐ll to be symptomatic, as they might have played a protective role, but this finding might be just coincidental.

In conclusion, we could not confirm whether the patient had this multiple endocrine immune dysfunction from BD as a coincidence or they had a common etiology. However, all these findings combined suggest that coincidental findings were extremely unlikely. These findings may also be the result from ceasing immunomodulation therapy in a patient with BD. We could not exclude PGA‐ll due to financial hurdles for genetic testing. Nevertheless, this was the first‐reported case of BD with AD, HD, and CD and further investigations are required.

## CONFLICT OF INTEREST

None declared.

## AUTHOR CONTRIBUTIONS

AK: M.D, Drafting, literature check, writing and revision, and final submission. RA: M.D, Acquiring and analyzing the data and final revision of the text.

## ETHICAL APPROVAL

Ethical approval was taken from Al Mouwasat University Hospital and Damascus University deanship.
